# 2299. Longitudinal Serological Surveillance of SARS-CoV-2 in Children and Adolescents Using Serial At-Home Testing

**DOI:** 10.1093/ofid/ofac492.137

**Published:** 2022-12-15

**Authors:** Amina Ahmed, Michael E DeWitt, Diane Uschner, William H Lagarde, DeAnna J Friedman-Klabanoff, Roberto P Santos, Michael A Gibbs, Hazel Tapp, Keerti L Dantuluri

**Affiliations:** Levine Children's Hospital at Atrium Health, Charlotte, North Carolina; Atrium Wake Forest Baptist Health/ Wake Forest University School of Medicine, Winston-Salem, North Carolina; The George Washington University, Rockville, Maryland; WakeMed Health and Hospitals, Raleigh, North Carolina; University of Maryland School of Medicine, Baltimore, Maryland; University of Mississippi Medical Center, Jackson, Mississippi; Atrium Health - Carolinas Medical Center, Charlotte, North Carolina; Atrium Health, Charlotte, North Carolina; Levine Children's Hospital at Atrium Health, Charlotte, North Carolina

## Abstract

**Background:**

Longitudinal serological surveillance is critical to understand dynamics of SARS-CoV-2 infections in children, a substantial portion of whom are asymptomatic. We describe trends in seroprevalence using at-home testing and evaluate demographic and clinical characteristics associated with seroconversion.

**Methods:**

Children 2–17 years old enrolled at 3 North Carolina sites (Figure 1) were followed April 2- December 31, 2021. Daily electronic surveys solicited symptoms and vaccination status. Four fingerprick lateral flow immunoassay tests were shipped to participants to be completed monthly; sensitivity and specificity were 84.5% and 99.0%, respectively.

We defined an infection window as 30 days before a positive antibody test (IgG), excluding results after any vaccine. Asymptomatic was defined as absence of “new” symptoms in the window; “new” was defined as not occurring the 14 days preceding the first observation of the symptom in the window. Univariate logistic regression was used to compare participants with and without infection-induced antibodies. For estimated seroprevalence, we used Bayesian inference accounting for sensitivity and specificity, modeling IgG positives from a binomial distribution.

**Results:**

Of 1,501 participants, 9.1% developed infection-induced antibody (Table 1). Blacks were more likely to seroconvert (OR 1.95 [95% CI 1.05–3.45]) as were those in a 5-or-more person household (OR 4.25 [CI 1.47–12.1]). Cumulative seroprevalence of SARS-CoV-2 increased from 12.7% in May to 32.4% in October (Figure 2); 61% of those seropositive were asymptomatic (Table 2). Adolescents had the highest seroconversion rates and were significantly more likely than 2–4-year olds to be asymptomatic.

Bayesian seroprevalence estimates accounting for assay sensitivity and specificity. Error bars represent 95% credible intervals.

**Conclusion:**

Prior SARS-CoV-2 seroprevalence data in children are limited by cross sectional design and use of convenience samples. With serial testing, we demonstrate rising SARS-CoV-2 seroprevalence, with highest rates of seroconversion in adolescents. Although prior findings suggest that asymptomatic infections occur most frequently in young children, we found a high proportion among adolescents. Our findings underscore the importance of serosurveillance to optimize public health efforts aimed at children and adolescents.

**Disclosures:**

**Roberto P. Santos, MD, MSCS**, American Board of Pediatrics - Sub-Board Infectious Diseases: Honoraria|Eli Lilly and Company: Grant/Research Support|Eli Lilly and Company: Eli Lilly and Company awarded to the University of Mississippi Medical Center with Roberto P. Santos as the Site PI|Health Resources and Services Administration (HRSA): Grant/Research Support|Health Resources and Services Administration (HRSA): Program Director & Principal Investigator|Janssen Pharmaceutical Companies of Johnson & Johnson (JNJ): Grant/Research Support|Janssen Pharmaceutical Companies of Johnson & Johnson (JNJ): Janssen Pharmaceutical Companies of Johnson & Johnson (JNJ) awarded to the University of Mississippi Medical Center with Roberto P. Santos as the Site|Merck Sharp & Dohme (MSD): Grant/Research Support|Merck Sharp & Dohme (MSD): Merck Sharp & Dohme (MSD) awarded to the University of Mississippi Medical Center with Roberto P. Santos as the Site PI.

**Figure 1.**
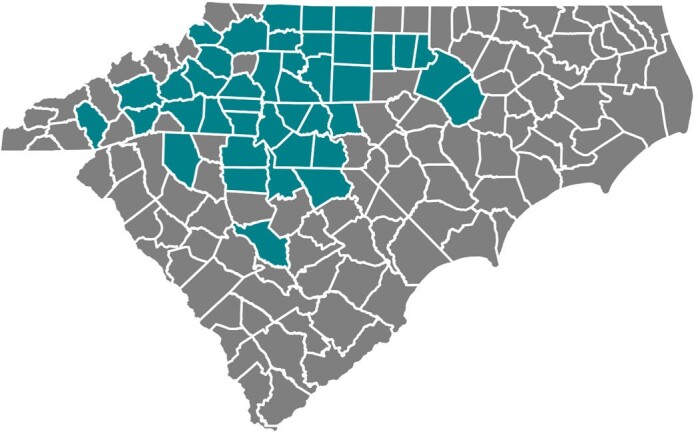
Participants' Counties of Residence in North and South Carolina

**Table 1.**
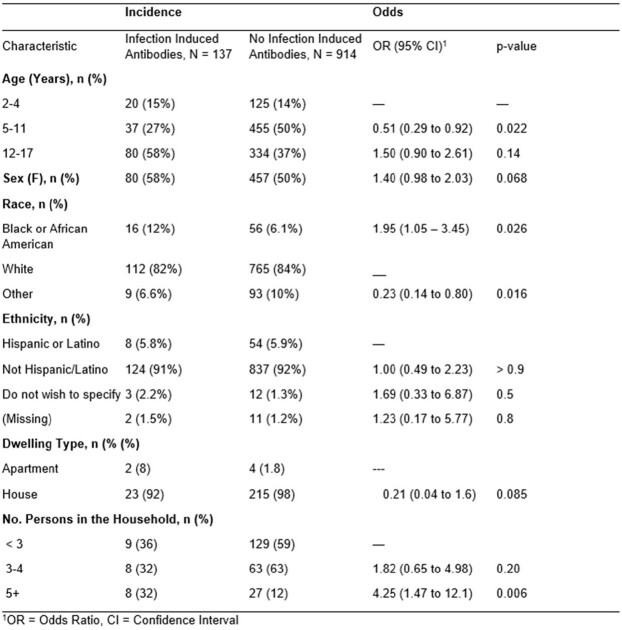
Demographic and Household Characteristics of Participants with and without Infection-induced Antibodies.

**Figure 2.**
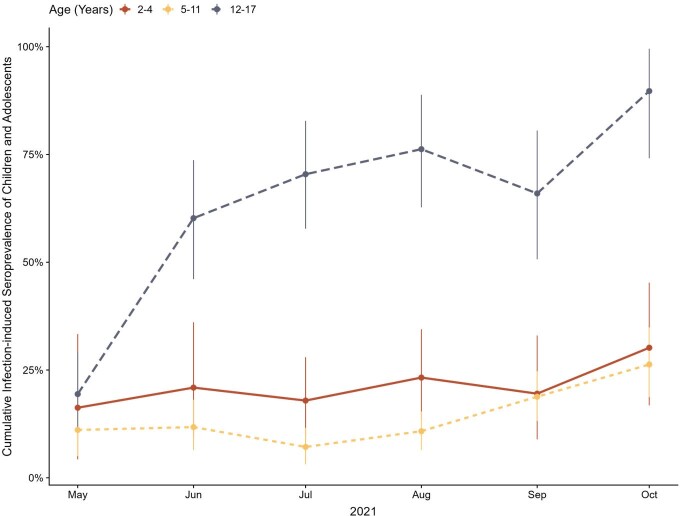
Cumulative Seroprevalence in Unvaccinated Children and Adolescents, May to October, 2021

**Table 2.**
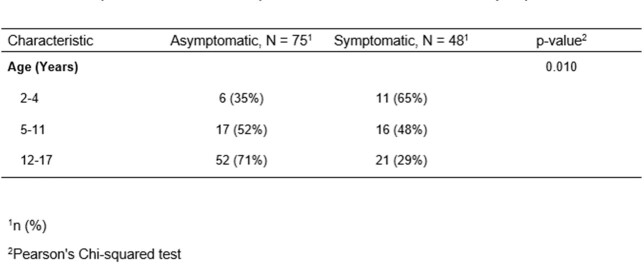
Proportion of Participants with and without Symptoms Prior to Seroconversion

